# Combined Impact of Magnetic Force and Spaceflight Conditions on *Escherichia coli* Physiology

**DOI:** 10.3390/ijms23031837

**Published:** 2022-02-06

**Authors:** Pavel A. Domnin, Vladislav A. Parfenov, Alexey S. Kononikhin, Stanislav V. Petrov, Nataliya V. Shevlyagina, Anastasia Yu. Arkhipova, Elizaveta V. Koudan, Elizaveta K. Nezhurina, Alexander G. Brzhozovskiy, Anna E. Bugrova, Anastasia M. Moysenovich, Alexandr A. Levin, Pavel A. Karalkin, Frederico D. A. S. Pereira, Vladimir G. Zhukhovitsky, Elena S. Lobakova, Vladimir A. Mironov, Evgeny N. Nikolaev, Yusef D. Khesuani, Svetlana A. Ermolaeva

**Affiliations:** 1 Laboratory of Ecology of Pathogenic Bactreia, Gamaleya National Research Centre for Epidemiology and Microbiology, 123098 Moscow, Russia; paveldomnin6@gmail.com; 2 Laboratory for Biotechnological Research «3D Bioprinting Solutions», 115409 Moscow, Russia; vapar@mail.ru (V.A.P.); 666online@mail.ru (S.V.P.); koudan1980@gmail.com (E.V.K.); levin.alexandr.stankin@gmail.com (A.A.L.); freddasp@gmail.com (F.D.A.S.P.); vladimir.mironov54@gmail.com (V.A.M.); usefhesuani@yandex.ru (Y.D.K.); 3 Center for Computational and Data-Intensive Science and Engineering, Skolkovo Institute of Science and Technology, 121205 Moscow, Russia; konoleha@yandex.ru (A.S.K.); agb.imbp@gmail.com (A.G.B.); E.Nikolaev@skoltech.ru (E.N.N.); 4 Gamaleya National Research Centre for Epidemiology and Microbiology, Laboratory of Indication and Ultrastructural Analysis of Microorganisms, 123098 Moscow, Russia; nataly-123@list.ru; 5 Biology Department, Moscow State University, 119991 Moscow, Russia; anastasia-yu.arkhipova@yandex.ru (A.Y.A.); a-moisenovich@mail.ru (A.M.M.); elena.lobakova@gmail.com (E.S.L.); 6 Biological Faculty, Shenzhen MSU-BIT University, Shenzhen 518172, China; 7 National Medical Research Radiological Centre, P. Hertsen Moscow Oncology Research Institute, 125284 Moscow, Russia; eliznezhurina@gmail.com; 8 Emanuel Institute of Biochemical Physics, Russian Academy of Sciences, 119991 Moscow, Russia; annabugrova@gmail.com; 9 Institute of Cluster Oncology named after Professor L.L. Levshin, I. M. Sechenov First Moscow State Medical University of the Ministry of Health, 127473 Moscow, Russia; pkaralkin@gmail.com; 10 Russian Medical Academy of Continuing Professional Education (RMANPO), Ministry of Public Health, 125993 Moscow, Russia; zhukhovitsky@rambler.ru

**Keywords:** space flight, bacterial metabolism, magnetic force, glyoxylate shunt, methylglyoxal bypass

## Abstract

Changes in bacterial physiology caused by the combined action of the magnetic force and microgravity were studied in *Escherichia coli* grown using a specially developed device aboard the International Space Station. The morphology and metabolism of *E. coli* grown under spaceflight (SF) or combined spaceflight and magnetic force (SF + MF) conditions were compared with ground cultivated bacteria grown under standard (control) or magnetic force (MF) conditions. SF, SF + MF, and MF conditions provided the up-regulation of Ag43 auto-transporter and cell auto-aggregation. The magnetic force caused visible clustering of non-sedimenting bacteria that formed matrix-containing aggregates under SF + MF and MF conditions. Cell auto-aggregation was accompanied by up-regulation of glyoxylate shunt enzymes and Vitamin B12 transporter BtuB. Under SF and SF + MF but not MF conditions nutrition and oxygen limitations were manifested by the down-regulation of glycolysis and TCA enzymes and the up-regulation of methylglyoxal bypass. Bacteria grown under combined SF + MF conditions demonstrated superior up-regulation of enzymes of the methylglyoxal bypass and down-regulation of glycolysis and TCA enzymes compared to SF conditions, suggesting that the magnetic force strengthened the effects of microgravity on the bacterial metabolism. This strengthening appeared to be due to magnetic force-dependent bacterial clustering within a small volume that reinforced the effects of the microgravity-driven absence of convectional flows.

## 1. Introduction

The prospect of future long-term space flights makes it necessary to study mechanisms of adaptation to microgravity not only by humans, but also by the organisms that will accompany them. Bacteria play a leading role in human health and are widely used in biotechnological applications. The development of antibacterial treatments in space flights, the search for novel biotechnological applications, and the improvement of bacterial growth conditions depend on understanding changes in bacterial physiology associated with spaceflight conditions [1,2,3].

Space studies performed aboard manned spacecrafts and Space Stations Mir and ISS (International Space Station) demonstrated that spaceflight conditions stimulated noticeable changes in bacterial morphology, stress tolerance, genomic stability, and virulence in pathogenic species [4,5,6,7,8,9,10]. These changes result from adaptation to specific environmental conditions of spaceflight, including microgravity, absence of gravity-driven convective flows, and presence of space radiation [11,12]. Besides these natural factors, specific problems of space flights and safety requirements put other restrictions on experiments with bacterial cultures, including gravitational overloads when bacteria are being delivered to the spacecraft and back to the Earth, and microaerophilic or anaerobic conditions due to hermetically sealed experimental flasks [6,9]. However, the increasing diversity of experimental settings allows better differentiation of unescapable influences associated with the space environment and avoidable restrictions dependent on particular experimental conditions.

In this work, we introduced the additional force, which was a magnetic force, to analyze changes in bacterial behavior under spaceflight conditions. All living creatures are diamagnetic. The magnetic force emerges due to the repelling of diamagnetic objects by a non-homogenous magnetic field [13,14,15,16]. If the magnetic force is directed opposite gravity, the resulting vector might reach a zero value, so the magnetic force compensates the gravity. This phenomenon is known as magnetic levitation [13].

Magnetic levitation is widely used in engineering and technology. The magnetic levitation approach was applied to animals, cells, and microorganisms, including bacteria [13,15,16,17,18]. Recently, Parfenov et al. described a magnetic levitation system that uses relatively low magnetic fields due to the supplementation of paramagnetic nutritive medium with the salt of the paramagnetic rare-earth element gadolinium [15]. Gadolinium-including compounds are used in MRI diagnostics and are non-toxic. Bacteria when grown under conditions of magnetic levitation in the medium supplemented with the gadolinium derivative gadobutrol (Gd-DO3A-butrol) form levitating aggregates, which consist of cells, an extracellular-matrix, and resemble well-known biofilms aside from the fact that the levitating aggregates are not attached [16].

At spaceflight conditions, the magnetic force has nothing to counterbalance, although it is still a force driving diamagnetic objects into the direction of the lowest magnetic field. In this part, experimental conditions are different from standard microgravity conditions when there are no forces to push bacterial cells. So, the introduction of the magnetic force into the spaceflight experiment might change bacterial behavior. Our study used the potential of the gadolinium-based magnetic levitation system to explore changes introduced in bacterial behavior under spaceflight conditions by the magnetic force. Bacterial behavior under combined microgravity and a magnetic force conditions was compared with bacterial behavior under conditions of pure microgravity and ground magnetic levitation.

## 2. Results

### 2.1. Experimental Design

**Figure 1:**
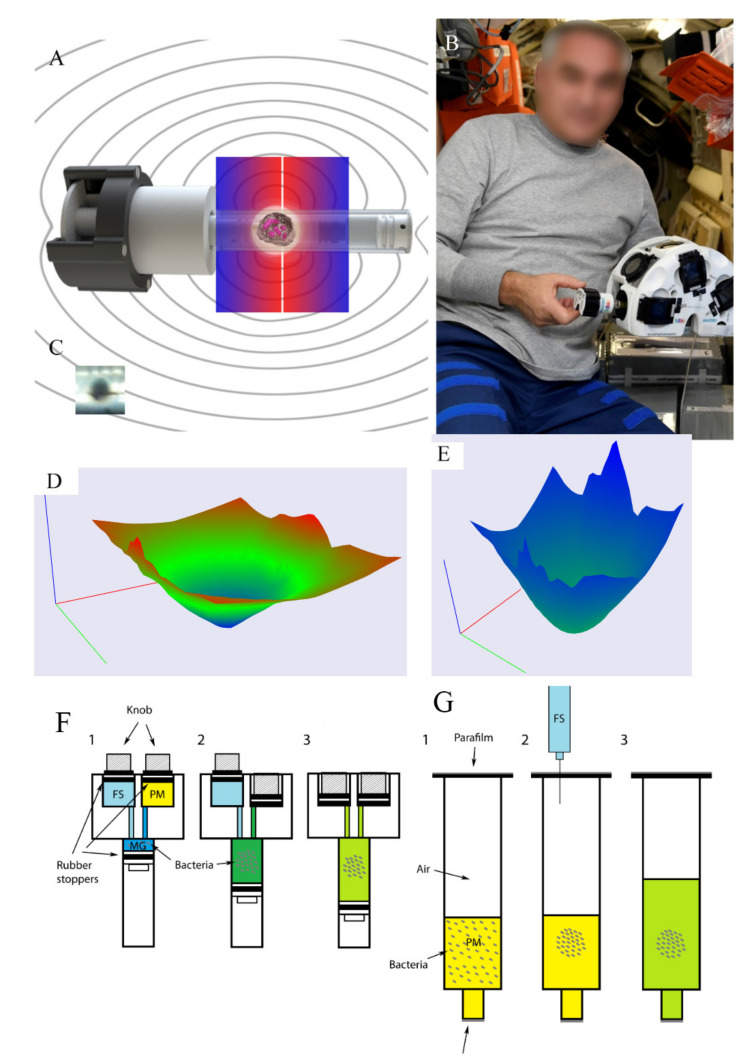
The design of the experiment: (**A**) the bioassembler «Organ.Aut» unit providing the magnetic field gradient; (**B**) the cosmonaut of Roscosmos, performing the experiment aboard the ISS; (**C**) macroaggregate formed under the magnetic force; (**D**) distribution of the magnetic flux density in the working volume, B_max_ = 0.7682 T, B_min_ = 0 T; (**E**) distribution of the magnetic force affecting the bacterium within the working volume, Fm_max_ = 2 × 10^−10^ N, Fm_min_ = 0; (**F**) the hardware used in the spaceflight experiment; 1: before the start of the experiment bacteria were placed in the Melbiol hydrogel (MG), the nutritive paramagnetic medium (LB + 1 M Gadovist, PM) and fixation solution (FS) were in the isolated chambers; 2: then the cosmonaut pushed a button to mix MG and PM, and bacteria started to grow; 3: at the end of the experiment, the cosmonaut pushed a second button to fix bacteria; the total system hermetically sealed without air bubbles; (**G**) the hardware used in the ground experiment; 1: bacteria were diluted with the paramagnetic medium (PM); 2: bacteria were grown for 144 g under conditions of magnetic levitation before the fixation solution was added by angling with a syringe; 3: fixed bacterial culture.

Bacteria grown aboard the International Space Station (ISS) were placed into the bioassembler «Organ.Aut» (Figure 1). The bioassembler included six independent sockets, each with two permanent magnets that provided a constant magnetic field gradient. The magnetic gradient values were the same in the space bioassembler «Organ.Aut» and the ground bioassembler (Figure 1 and Figure S1).

To get a high magnetic force, a higher difference between diamagnetic properties of cells and medium was reached by using a paramagnetic medium, which was the LB broth supplemented with 20% Gadovist^®^. Gadovist^®^ is a 1M salt of the rare-Earth element gadolinium that possesses paramagnetic properties. The magnetic force that affected bacteria grown in the paramagnetic medium was F_m_ ~ **B** × grad**B**, where B is a magnetic flux density at a given point of the working volume, and grad**B** is its gradient at the same point (see Materials and Methods for details). Therefore, the force F_m_ changed from higher values near magnets to a zero value in the center of the working volume. Using a numerical approach, the force F_m_ was calculated for every point of the space within the working volume (F_max_ = 2 × 10^−10^ Н, F_min_ = 0 Н; Figure 1). However, being placed in the bioassembler bacteria grew in a relatively small volume near the center where the magnetic force counterbalanced gravity that provided an effect of the magnetic levitation (MF conditions) (Figure S1). The magnetic force F_m_ that affected the bacterium in these stationary conditions was F_m_ = (3 ÷ 60) × 10^−17^ N (see Suppl. File S1).

The probiotic *E. coli* strain M17 was used in all experiments. Bacteria were grown for 144 h for all but one experiment. This time was chosen on the basis of previously described results: the 144 h growth allowed the formation of an easy-to-manipulate aggregate under magnetic levitation conditions [16]. The only exception was the ground control (24 h control), which included bacteria grown in the LB broth without agitation for 24 h that provided standard stationary culture. Another ground control included the culture grown in the LB broth supplemented with 20% PBS without agitation for 144 h (144 h control). The control that tested an effect of the magnetic force underground conditions included bacteria grown in the LB broth supplemented with 20% Gadovist^®^ (the paramagnetic medium) in the ground bioassambler (Figure S1; MF conditions). Bacteria placed into the «Organ.Aut» at the ISS board were grown in the same paramagnetic medium as described (SF + MF conditions) or in the LB broth supplemented with 20% PBS (SF conditions). The scheme of the experiment is shown at the Table S1.

Being grown under SF + MF conditions, bacteria formed a macroscopic structure of about 2 mm diameter at the area of the lowest magnetic field. Similar macroscopic structures were observed under conditions of magnetic levitation when the magnetic force counterbalanced gravity in ground experiments [16] (also see Figure S1). Spaceflight conditions (SF) themselves without the magnetic force did not cause visible macroscopic changes in bacterial distribution over the volume.

**Figure 2:**
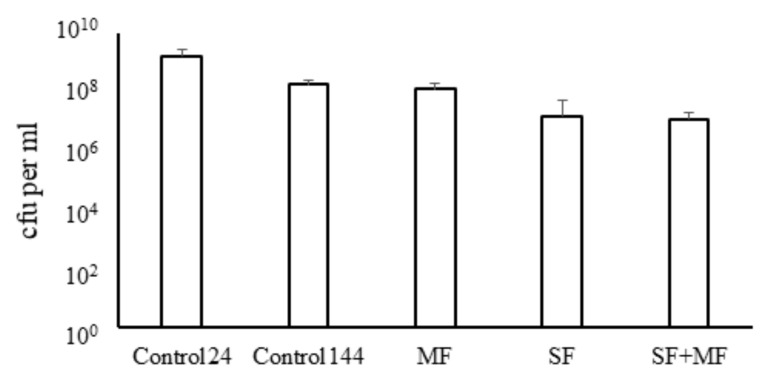
Bacterial counts. Bacteria were grown with shaking for 24 h (Control 24) or without shaking for 144 h under the following conditions: under ground conditions (Control 144); under ground magnetic force (MF) conditions; under spaceflight (SF) conditions; under combined spaceflight and magnetic force (SF + MF) conditions; serial dilutions were plated to count bacteria after space samples were delivered to the Earth.

The absolute counts of bacteria were determined by direct plating serial dilutions of alive non-fixed samples delivered to the Earth (Figure 2). Counts of bacteria grown under SF and SF + MF conditions differed non-significantly (*p* = 0.06) and were about 100-fold lower than in the overnight ground culture (*p* < 0.01). Bacteria grown on Earth with or without MF for 6 days (144 h) showed about 10-times higher counts comparatively with bacteria grown under SF and SF + MF conditions but about 10-times lower than the 24 h ground control (*p* < 0.05).

### 2.2. Changes in Morphology Caused by Spaceflight and a Magnetic Force

**Figure 3:**
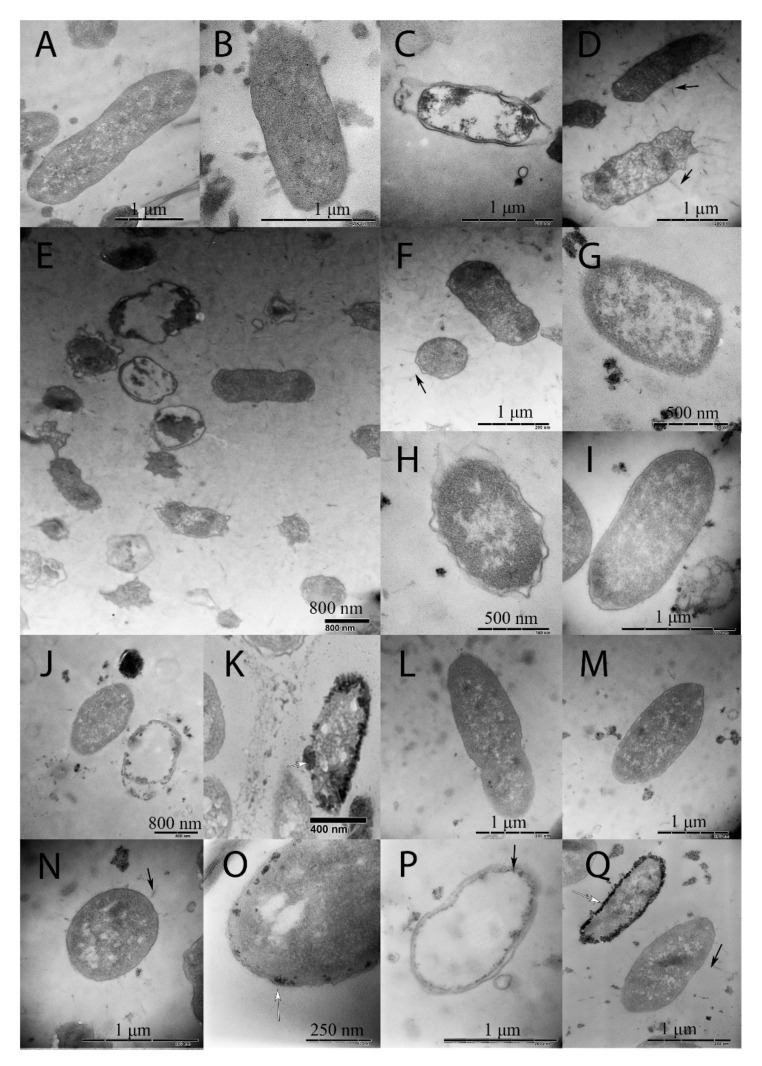
Transmission electron microscopy of E. coli M 17 grown underground and spaceflight conditions. (**A**) 24 h control; (**B**,**C**) 144 h control; (**D**–**F**) 144 h MF conditions; (**G**–**K**) 144 h SF conditions; (**L**–**Q**) 144 SF + MF conditions. Arrows show flagella. Electron-dense inclusions characteristic foe the culture grown in spaces are visible at (**K**,**O**,**Q**).

In 144 h cultures, TEM revealed both intact and damaged cells in all samples, including the 144 h control ground samples (Figure 3B–Q). Observed changes in damaged cells included defects in the cell wall structure and signs of lysis up to almost lysed cells. There were no specific damages characteristic for spaceflight cultures only.

The specific feature of bacterial cultures grown under spaceflight conditions was the appearance of electron-dense small inclusions on the cell periphery observed in a part of the bacterial population grown at the ISS board (Figure 3K,O,Q). These inclusions resembled previously described outer membrane vesicles (OMVs) formed in *E. coli* under spaceflight conditions [4]. Such inclusions were not observed in ground cultures, including the culture grown underground in MF conditions (Figure 3D–F).

Spaceflight conditions caused bacterial cell shortening comparatively to the 24 h control ground culture, the difference between SF and 144 h control ground cultures was insignificant (3.0 µm vs. 3.72 µm for SF and 24 h cultures, respectively, *p* < 0.05; 3.0 µm vs. 3.27 µm for SF and 144 h cultures, *p* = 0.49: Figure 3 and Table 1). The introduction of the magnetic force prevented cell shortening under SF conditions (3.64 µm vs. 3.27 µm for SF + MF and 144 h grounds conditions, respectively; *p* < 0.5). Interestingly, that the magnetic levitation in ground conditions (ML conditions) provided the highest shortening effect in comparison with all conditions tested (1.92 µm vs. 3.27 µm for MF and 144 h ground cultures, respectively; *p* < 0.05).

### 2.3. Aggregates Formed by Cells and Non-Cellular Matrix under SF + MF Conditions

**Figure 4:**
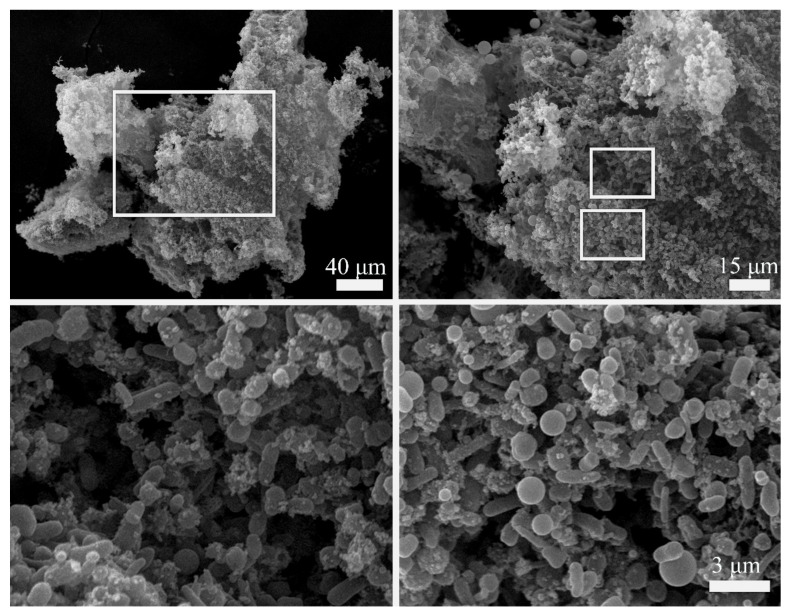
Aggregates formed by bacteria and extracellular matrix. Bacteria were grown under SF + MF conditions for 144 h, then fixed and studied with scanning electron microscopy. The rectangles show enlarged areas.

Scanning electron microscopy of samples grown under SF + MF conditions demonstrated that the observed macroscopic structures were formed by bacterial aggregates that included bacteria and non-cellular components (Figure 4). The non-cellular components included bubble-shaped structures together with less-structured substances. At the same time, non-structured substances or tiny bubbles might be matrix components produced by bacteria themselves. The bubble-shaped structures with a diameter similar or noticeably more significant than a bacterial cell might be artifacts formed by added substances such as a hydrogel rather than cells products.

### 2.4. Proteomic Studies

Proteomic analysis of *E. coli* M17 revealed 23 proteins that changed expression depending on spaceflight conditions and/or introduction of a magnetic force compared to the ground control (in two replicas, 1.5-fold threshold; *p* < 0.05). Functional annotation based on the KEGG database showed that differentially expressed proteins belonged to several functional classes, including genetic information processing, carbohydrate and nucleotide metabolism, stress response, and surface structures.

#### 2.4.1. SF vs. Ground Control

Changes in genetic information processing proteins, particularly down-regulation of the RNA-polymerase β’-subunit RpoB, suggested that transcription was restricted in the SF-grown culture (Table 2). In the meantime, the ribosomal proteins manifesting translation activity changed insignificantly, which suggested that some stable RNAs were effectively translated under SF conditions.

Five stress response proteins changed expression under SF conditions. The oxidative stress response proteins hydroperoxidase KatG and NAD(P)H-quinone) dehydrogenase WbrA (NqoR) were up-regulated, suggesting that prolonged growth under spaceflight conditions resulted in the accumulation of toxic by-products. In contrast, chaperones DnaK and ClpB and the ClpP subunit of the protease ClpXP responsible for proteolytic degradation of misfolded proteins were down-regulated.

Changes in carbohydrate metabolism suggested that SF conditions were mainly anaerobic while bacteria were starving. The anaerobic metabolism was manifested by the up-regulation of isocitrate lyase AceA and down-regulation of isocitrate dehydrogenase Idh that suggested glyoxylate shunt activation on the background of the tricarboxylic acid (TCA) cycle repression. Down-regulation of pyruvate formate lyase 4 TdcE and pyruvate formate-lyase PflB suggested inhibition of glycolysis that appeared to be caused by starvation. Up-regulation of the methylglyoxal synthase MgsA suggested upregulation of the methylglyoxal pathway. The methylglyoxal is a toxic intermediate product that is converted into pyruvate via D-lactate. In this way, the methylglyoxal pathway is considered as an alternative to glycolysis.

Among changes in surface structures, up-regulation of the autotransporter protein Antigen 43 was observed. Ag43 is involved in cell auto-aggregation [19]. The phenomenon of auto-aggregation is well documented for bacteria growing under microgravity conditions [4,19]. In addition, a noticeable 16-fold up-regulation of Vitamin B-12 transporter BtuB was observed, suggesting cell requirements for Vitamin B-12 dependent proteins, ethanolamine deaminase and/or methionine synthase in *E. coli* culture.

#### 2.4.2. SF + MF vs. Ground Control and SF

Changes in protein abundance under SF + MF conditions and SF conditions compared to the ground control suggested that the magnetic force strengthened trends associated with the SF conditions. Comparatively to the ground control, KatG was up-regulated 2.0- and 2.8-fold, WrbA was up-regulated 2.8- and 4.0-fold, DnaK was down-regulated 3.0- and 7.5-fold, under SF and SF + MF conditions, respectively (Table 2). In addition, SF + MF conditions provided a more severe effect on protein transcription and translation than SF conditions.

The carbohydrate metabolism moved to strict anaerobiosis under SF + MF conditions. The shift to anaerobiosis was manifested by (i) three-fold down-regulation of transketolase Tkt1; (ii) further down-regulation of pyruvate formate-lyase PflB (3.0- and 5.3-fold reduction under SF and SF +MF conditions, respectively, relative to the ground control); (iii) 2.8-fold up-regulation of anaerobic glycerol-3-phosphate dehydrogenase GlpA. GlpA converts glycerol-3-phosphate to dihydroxyacetone phosphate (DHAP) using fumarate as a terminal electron acceptor [20]. Further transversion of DHAP causes accumulation of methylglyoxal, supporting activation of the methylglyoxal pathway. On the whole, these results suggested that the magnetic force enhanced the anaerobic effects observed under spaceflight conditions.

#### 2.4.3. MF vs. Ground Control

To understand whether the observed enhancement of anaerobiosis under SF + MF conditions comparatively to SF conditions was due to a specific influence of the magnetic field, we compared proteins of bacteria grown on the Earth under magnetic force (MF) conditions with the ground control (Table 2). This comparison demonstrated that the magnetic field itself did not stimulate manifestations characteristic of a strict anaerobic response. Although the glyoxylate shunt was activated under MF conditions as manifested by 2.5-fold AceA up-regulation, this activation was not accompanied by down-regulation of the TCA cycle and Isocitrate dehydrogenase Idh was up-regulated 3.7-fold in contrast to its two-fold down-regulation under SF conditions. Both methylglyoxal synthase MgsA and anaerobic glycerol-3-phosphate dehydrogenase GlpA were down-regulated 2.0- and 2.6-fold under MF conditions compared to the control suggesting down-regulation of the methylglyoxal shunt in contrast to SF and SF + MF conditions. Changes in stress response proteins under MF conditions were different from those observed under SF and SF + MF conditions. While SF and SF + MF conditions caused down-regulation of all the heat shock family proteins, MF conditions caused the reverse effects in chaperones and heat shock proteases. Under MF conditions, the chaperone ClpB was two-fold up-regulated, while the heat shock protease ClpX was two-fold downregulated. Therefore, obtained results suggested that the magnetic field was not responsible for changes observed under SF + MF conditions.

#### 2.4.4. SF vs. MF

At last, we compared proteins patterns under spaceflight SF and ground MF conditions. Observed differences in carbohydrate metabolism supported the view that experimental settings of the spaceflight experiment rather than the magnetic field were responsible for anaerobic cell response on SF + MF conditions. The proteins involved in the anaerobic response were up-regulated, while proteins involved in the TCA cycle and glycolysis were down-regulated under SF conditions comparatively to MF ground conditions. Oxygen stress proteins were up-regulated, while chaperone proteins were down-regulated under SF vs. MF conditions. The most significant difference was observed in flagellin FliC protein, which was 14-fold down-regulated by SF conditions. Still, some similar changes were observed under SF and MF relatively to the ground control that included activation of the glycosylate shunt and up-regulation of the Ag43 and BtuB surface proteins.

## 3. Discussion

Here we studied changes in morphology and physiology of the *E. coli* strain M17 grown under conditions of spaceflight coupled with the constant magnetic force. The most prominent feature of the spaceflight (SF) conditions is microgravity associated with low shear fluid dynamics and the absence of convection currents [1,2,21]. Many studies demonstrated that bacteria show a species- and strain-specific response to microgravity, including changes in morphology, physiology, metabolism, and virulence [4,5,6,7,10,22,23,24].

The specific feature of our research was the introduction of the constant magnetic force (MF) into the spaceflight experiment (SF + MF conditions). The present study has shown that the presence of a magnetic force under spaceflight conditions alters the morphology and physiology of bacteria compared to microgravity alone. Some of these changes strengthened SF effects, while others were opposed to SF effects.

**Figure 5:**
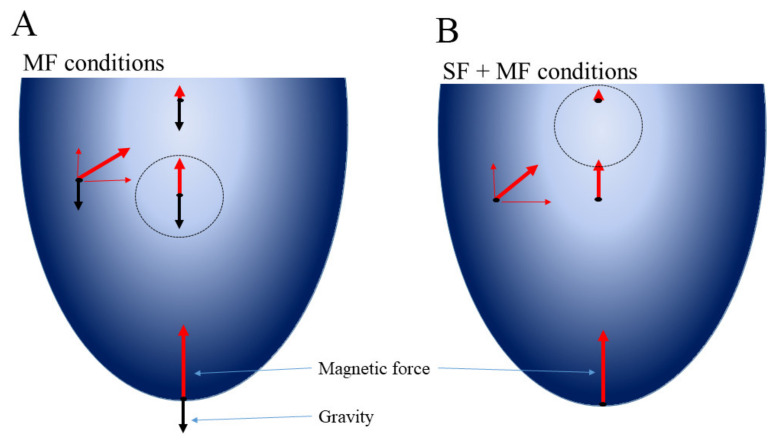
Forces that affected bacteria under MF (**A**) and SF + MF conditions (**B**). Red arrows—the magnetic force; black arrows—the gravitational force. The Archimedes force is not shown to simplify the figure (for details see the Supplementary File S1). The gradient of the blue represents the magnetic field strength that decreases as the distance from the magnets (the walls) increases. The circles designate the area with the lowest resultant force where bacteria are concentrated.

Below, we discuss the observed changes. Here we would like to specify physical forces affecting bacteria under spaceflight and ground conditions when the magnetic force is introduced. In underground conditions, bacteria are affected by a resultant force comprised of a vector sum of gravitational, Archimedes and magnetic forces (Figure 5). This resultant force moves bacteria to the area where forces counterbalance each other and that results in magnetic levitation. In the absence of gravity, the magnetic force was the only force that affected bacteria (Figure 5). Therefore, under SF + MF conditions, bacteria were driven to the area with the lowest magnetic field. The presence of the magnetic force made the SF + MF conditions different from SF conditions, where there were no forces affecting bacteria.

### 3.1. Reduced Bacterial Growth

Increased bacterial proliferation and higher final cell densities under spaceflight conditions have been reported by many researchers [4,12,25]. Still, some studies did not reveal noticeable changes in growth [26,27]. Theoretical analysis performed by Klaus and colleagues suggests that the outcome of bacterial growth under microgravity conditions depends on a ratio of the positive effect of better availability of nutrients for non-sedimenting bacteria, to the negative effect of toxic by-product accumulation in the vicinity of the non-sedimenting bacterium [12]. When exponentially growing or when early stationary cultures are studied in spaceflight or modeled microgravity conditions, positive effects prevail, resulting in a noticeable (sometimes more than 10-fold) improved cell outcome [4,12]. However, the situation is different upon prolonged culture incubation as the accumulation of toxic by-products in the vicinity of the bacterium might cause the death [12].

After six days of incubation, we observed a decrease in viable cell counting in the cultures grown under SF and SF + MF conditions. It was in line with the theoretical predictions of the decrease in bacterial population due to the accumulation of toxic by-products. Methylglyoxal might be one of such by-products. The proteomic research revealed the up-regulation of proteins involved in the synthesis of methylglyoxal (see Table 2). Methylglyoxal, known as 2-oxopropanal or pyruvaldehyde, is a highly cytotoxic physiological metabolite that can glycate and crosslink macromolecules like proteins and DNA [28]. Methylglyoxal can be secreted and accumulated outside the cell, although, under standard conditions, *E. coli* usually secreted low methylglyoxal to detect. On the other hand, we cannot entirely exclude the possibility that the reduced bacterial counts might be due to the mortality of a part of the population during delivery to the Earth.

### 3.2. Role of Cobalamin Outer Membrane Transporter BtuB for Prolonged Survival under SF, SF + MF, and Ground MF Conditions

The critical feature differentiating bacteria grown under SF/SF + MF and MF conditions from the 144 h control was the over-expression of the cobalamin outer membrane transporter BtuB. In *E. coli*, there are at least two vitamin B12-dependent enzymes, which are the B12-dependent methionine synthase and ethanolamine deaminase [29]. The B12-dependent methionine synthase that catalyzes the last step in methionine biosynthesis has a functional homolog independent of B12, so the necessity of the cobalamine-dependent protein could not be so high [30]. The ethanolamine deaminase is used to utilize ethanolamine, a valuable microbial carbon, and nitrogen source derived from the membrane phospholipid phosphatidylethanolamine [31]. Cell membranes could be supplied by lysed cells, and BtuB up-regulation might be required to provide effective utilization of a dead part of the population. In the control 144 h ground culture dead bacteria sank to the bottom. However, the situation was different in cultures grown under SF/SF + MF and MF, where dead and alive bacteria were in close vicinity. So, membranes supplied by lysed cells should be in excess in cultures grown under SF/SF + MF and MF conditions, explaining the requirements for BtuB.

### 3.3. Changes in Bacterial Sizes

Many works reported species-specific morphological changes in bacteria grown under spaceflight conditions [4,5,7,25]. As such, Zea and colleagues reported a 37% decrease in the cell volume thickening of the cell wall and the accumulation of secreted vesicles for the non-motile *E. coli* strain ATCC4157 [4]. However, some other studies did not reveal noticeable changes in *E. coli* cell and cell wall parameters [7,25]. In our research, while bacteria grown under SF conditions were shortened comparatively to the standard 24 h culture, we did not observe a statistically significant difference in the cell length when parallel 144 h ground and SF cultures were compared. Interestingly, the magnetic force provided an alternative effect on bacterial length on the ground and in space. While bacteria grown under MF conditions were shorter than the 24 h and 144 h controls and SF samples, the length of bacteria grown under SF + MF conditions was similar to the 24 h control. Thus, we can speculate that the magnetic force introduction in some way mimics gravity under spaceflight conditions, while under MF conditions, bacteria underwent a zero pushing force. In other words, microgravity provides an essential effect on the cell length, and the introduction of the magnetic force into the spaceflight conditions might disturb this effect.

### 3.4. Bacterial Autoaggregation

Microgravity stimulates cell auto-aggregation and clamping. Zea et al. demonstrated cell clustering on the non-motile *E. coli* grown at the ISS board [4]. The comprehensive experiment on *Salmonella typhimurium* grown on the board of on Space Shuttle Atlantis Mission STS-115 demonstrated that bacterial aggregates include extracellular matrix and the consistent up-regulation of some genes involved in the matrix production course of formation of biofilms [7]. Auto-aggregation was demonstrated for different bacterial species grown under modeled microgravity conditions [32,33]. The introduction of the magnetic force made auto-aggregates to form macroscopic structures. The autoaggregates included bacteria and an extracellular matrix (see Figure 4). Similar auto-aggregates were observed under magnetic levitation conditions [16].

### 3.5. Role of Low Nutrient and Oxygen Availability and Carbohydrate Metabolism under Spaceflight Conditions

The particular characteristic of our research was prolonged bacterial incubation for six days. The duration was determined by previous experiments with magnetic levitation, which demonstrated that the maturation of levitating aggregate requires about 5–6 days [16]. This time corresponds to the late stationary phase of the liquid ground culture when nutritive resources are exhausted, cells starve, and survivors recycle resources released by lysed cells [34].

**Figure 6:**
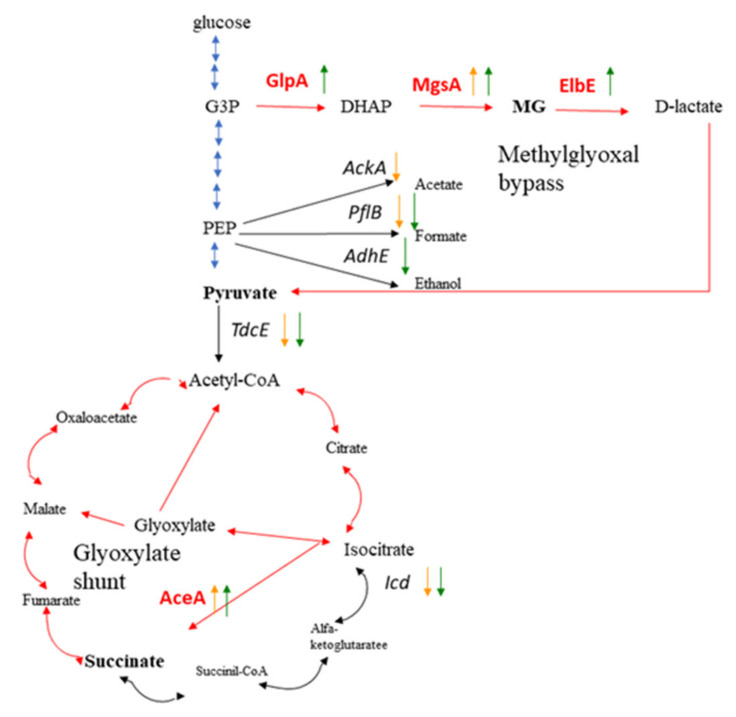
Metabolic pathways up- and down-regulated under spaceflight conditions. Up-regulated pathways and enzymes are shown by red; down-regulated pathways and enzymes are shown by black; other pathways are shown by blue. Vertical arrows show directions of enzyme regulation: orange arrows related to SF conditions; green arrows related to SF + MF conditions. Metabolites discussed in the text are highlighted with bold font.

Two pathways were up-regulated in cells grown under SF and SF + MF conditions; these pathways were the methylglyoxal bypass and the glyoxylate shunt (Figure 6). Nutrition limitations, availability of direct glycolysis intermediates and other metabolites supplied by lysed cells, as well as oxygen limitations, might result in the methylglyoxal bypass and the glyoxylate shunt activation under SF and SF + MF conditions.

The methylglyoxal bypass is activated under conditions of nutrition limitations or excess of direct glycolysis intermediates [28,35,36,37]. Conversion of methylglyoxal into pyruvate via D-lactate is a preferred direction for pyruvate generation in conditions of glycolytic impediment [28,38]. The glyoxylate shunt shares the activities of some enzymes with the TCA cycle. However, it differs by the shortcut to convert isocitrate into succinate via isocitrate lyase AceA and malate via glyoxylate due to malate synthase AceB. The glyoxylate shunt is essential when acetyl-CoA is a direct product of a metabolic pathway, for example, via degradation of acetate, fatty acids, and alkanes, i.e., this pathway allows cell survival under starvation and enables cells to utilize C2 units more efficiently for biomass production [39,40]. The net reaction of the glyoxylate shunt allows cells to convert two acetyl-CoA units into succinate and avoid the CO2-releasing steps of the TCA cycle [41]. Thus, activation of the glyoxylate shunt is more effective in terms of biomass production and allows a decrease in environmental acidification that is important for the cell in the absence of convectional flows. Succinate, which is the glyoxylate shunt product, comprises the H+ acceptor under anaerobic conditions instead of low available oxygen [42]. Under aerobic conditions, the production of succinate is not naturally possible. Therefore, changes in carbohydrate metabolism suggested that bacteria under SF and SF + MF conditions underwent nutrient and oxygen limitations, and the metabolism of survivals was changed to provide adequate consumption of components available from lysed cells.

Using transcriptome and proteome approaches, Crabbe et al. demonstrated an anaerobic mode of growth for *P. aeruginosa* PAO1 under spaceflight conditions and suggested the fluid-processing apparatus (FPA) design that provided the limited oxygen availability for bacteria grown at the ISS board [6]. The hardware used in our spaceflight experiment was very similar to the FPA described by Crabbe et al. (see Figure 1); thus, our results supported a severe bacterial response to low oxygen availability under spaceflight conditions that could be partly due to the experimental design.

### 3.6. Effect of the Magnetic Force on Cell Response on Oxygen and Nutrient Limitations

The comparison of SF and SF + MF conditions demonstrated that the introduction of the magnetic force further strengthened the response to oxygen and nutrient limitations. All enzymes involved in the methylglyoxal bypass and the glyoxylate shunt were more active under SF + MF conditions than SF conditions, while the impediment of glycolysis and the TCA cycle was more evident. For instance, the up-regulation of the anaerobic dehydrogenase GlpABC involved in the production of the methylglyoxal precursor DHAP from glycerol-3-phosphate (G3P) was observed under SF + MF conditions that suggested severe oxygen limitations [42].

The magnetic force’s effect on strengthening oxygen and nutrition limitations could be due to bacterial clustering in a smaller volume, determined by areas of the lowest magnetic field. In addition, the absence of convection currents in microgravity prevented oxygen and medium compound interchange between bacterium-containing and bacterium-free spaces that resulted in higher requirements in enzymes involved in survival under anaerobic and nutrition-limiting conditions. Parallel ground experiments on bacteria grown under MF conditions allowed evaluation of input of different parameters on changes in carbohydrate metabolism. Underground MF conditions, oxygen was provided by the presence of air in flasks that were half-filled with the medium (See Figure 1). Meanwhile, nutrition limitations were similar both in underground and in spaceflight conditions. The glyoxylate shunt activated in bacteria grown under MF as well as SF and SF + MF conditions, although the effect under MF conditions was not as pronounced as under SF/SF + MF conditions. Meanwhile, glycolysis impediment and upregulation of the methylglyoxal bypass were observed under SF and SF + MF conditions, however not under MF conditions, suggesting that oxygen limitations were more responsible for these changes in carbohydrate metabolism under SF conditions than effects of nutrition limitations, the magnetic force, or microgravity.

Overall, the obtained results demonstrated that oxygen and nutrient limitations under spaceflight conditions caused a bacterial response via the same mechanisms extensively studied on the Earth. Still, the absence of macroscopic convection currents in microgravity affects this response and the introduction of the magnetic force further strengthens it. These findings might be useful in biotechnology for the more effective production of certain compounds, such as succinate, which is widely used in chemical industry, particularly for synthesizing 1,4-butanediol based polymers, or methylglyoxal, which is used in oncology [43].

### 3.7. Outer Membrane Vesicles, Heat-Shock Protein Downregulation, and Misfolded Protein Accumulation Stress Response

A vital characteristic demonstrated by TEM was the appearance of electronically dense structures at the cell periphery in bacteria grown under spaceflight conditions. Similar structures were previously described by Zea and colleagues in *E. coli* grown under spaceflight conditions in the presence of gentamycin [4]. Such structures seem to represent outer membrane vesicles (OMVs) used to transport proteins and small molecules [4,44]. Production of outer membrane vesicles by Gram-negative bacteria was shown to be a specific stress response on the accumulation of misfolded proteins [44]. Considering the observed down-regulation of heat-shock proteins, the vesiculation process can be actual for misfolded protein removal under SF and SF + MF conditions.

### 3.8. Role of the Autotransporter Ag43

Proteome data demonstrated the up-regulation of Ag32 proteins in cultures grown under SF conditions, SF + MF, and MF conditions. The surface-displayed autotransporter protein Antigen 43 (Ag43) is responsible for *E. coli* auto-aggregation and flocculation in static liquid cultures [19,45]. Ag43 controls auto-aggregation and colony morphology in a phase-variable and stress-dependent manner [19,46]. Ag43 upregulation was shown to promote the cell aggregation via direct Ag43-Ag43 contacts under conditions of cell stress caused by the accumulation of misfolded protein in the cytoplasm [46].

Our results suggest that Ag43 is responsible for *E. coli* auto-aggregation under spaceflight conditions. The magnetic force applied to bacteria grown under spaceflight conditions did not further up-regulate Ag43 expression. Underground conditions, the magnetic force induced Ag43 production comparatively to the ground control up to the levels similar to the level observed under spaceflight conditions. We suppose that the Ag43 driven auto-aggregation plays an important role in metabolic changes under MF, SF, and SF + MF conditions. In particular, the Ag43-driven tight gathering of bacteria in auto-aggregates provided alive bacteria with materials from dead bacteria, which explains the up-regulation of glyoxylate shunt enzymes and Vitamin B12 transporter BtuB required to utilize metabolites from destroying cells. The situation was different in 144 h control when dead bacteria covered the bottom of the flask, while alive bacteria were swimming above. Taken together, the obtained data suggest that the magnetic levitation might be helpful to simulate interactions between *E. coli* cells taken place under microgravity.

## 4. Materials and Methods

### 4.1. Bacterial Strain

The *E. coli* strain M17 was used as a model microorganism. M17 is a non-virulent, motile, biofilm-forming strain used as a commercial probiotic [47]. In-ground experiments, under magnetic levitation conditions, M17 forms non-attached aggregates [16]. At ground conditions, bacteria were routinely cultivated in the LB broth (Sigma-Aldrich, St. Louis, MO, USA) at 37 °C with shaking. Spaceflight experimental settings are below.

### 4.2. Spaceflight Experimental Settings

The bacterial overnight culture was washed, resuspended in the same volume of PBS (phosphate-buffered saline), and premixed with thermoreversible Mebiol hydrogel (Mebiol Inc., Kanagawa, Japan, 1:100 *v*/*v*). The Melbiol gel prevented undesirable preliminary growth and attachment to cuvette walls during delivery to the ISS. Experiments were performed using specially designed hermetically sealed cuvettes (Figure 1). Cuvettes charged with hydrogel premixed bacteria were delivered to the Russian segment of the ISS by the rocket “Soyuz-FG” and the spacecraft “Soyuz MS-11.” All experiments were performed during the Space Expedition “ISS 58-59” course in December 2018. At the start of the experiment, the cosmonaut pushed the first button on the cuvettes to inject the paramagnetic medium into the Mebiol gel with bacteria. The paramagnetic medium was the sterile LB broth supplemented with 20% Gadovist^®^ (Bayer Schering Pharma AG, Leverkusen, Germany). Gadovist^®^ is 1 M gadobutrol ([10-[2,3-dihydroxi-1-(hydroxymethyl)propil]-1,4,7,10-tetraazacyclododexan-1,4,7-triacetin(3-)-N1, N4, N7, N10,O1, O4, O7]gadolinium) applied for MRI. Then the samples were cooled down to 17 °C for 90 min in a temperature-controlled chamber, which provided the “gel-sol” phase transition of thermoreversible hydrogel, and placed into the custom-designed magnetic bioassembler Bioprinter «Organ.Aut» (Figure 1). Bioassembler were transferred into the temperature-controlled chamber and incubated at +37 °C. Bacteria in control cuvettes grew in LB without Gadovist, so they experienced the magnetic field gradient but not the magnetic force. The samples suggested for proteomic analysis and microscopy were fixed with 4% formalin or with 2.5% glutaraldehyde, respectively, by pressing the second button on the cuvettes, and stored at room temperature for 26 days until return to Earth. The samples suggested for a direct plating were incubated for six days and then transferred to 4 °C. At this temperature, bacteria maintained for 21 days (17 days until return to Earth and four days to deliver from the Baikonur Cosmodrome (Kazakhstan) to Moscow (Russia).

### 4.3. Ground Control Experimental Settings

Overnight bacterial culture was diluted 1:100 with the paramagnetic medium and grown without agitation at 37 °C for 24 h or 144 h (six days), then bacteria were fixed and treated similarly to the spacecraft samples. Magnetic levitation experiments were performed with the previously described version of the bioassembler [16]. Generally, experiments were performed as previously described. Overnight bacterial culture was diluted 1:100 with a 2 mL paramagnetic medium, transferred to the 5 mL syringe sealed with Parafilm^®^, placed into the magnetic bioassembler, and put into the temperature-controlled chamber (37 °C). After six days of incubation, samples were fixed as described above.

### 4.4. Sample Preparation for Proteomic Analysis

Sigma-Aldridge (St. Louis, MO, USA) reagents were used for sample preparation unless otherwise noted. The fixed samples were centrifuged at 20,000× *g* at 4 °C for 20 min. The supernatants were discarded, the pellets were dissolved in 200 μL of lysis buffer with urea (8 M Urea / 1% NP40) and incubated with constant mixing for 30 min, centrifuged at 20,000× *g* at 4 °C for 20 min. The urea supernatants were collected. The pellets were re-extracted using SDS lysis buffer (4% SDS, 100 mM Tris-HCl, pH 8.0, 100 mM DTT) and incubated for 20 min at 100 °C, 2 h at 60 °C, sonicated for 40 min and centrifuged at 20,000× *g* at 4 °C again. The urea and SDS supernatants were pooled, the protein content was determined by the BCA assay (ThermoFisher Scientific, Waltham, MA USA). Proteins were diluted with 8 M urea/100 mM Tris-HCl, pH 8.0/100 mM DTT, and reduced for an hour at 37 °C. Filter-aided sample preparation (FASP) was performed according to the protocol (Wishnevsky, 2016) using 10 kDa Microcon filter units (Millipore, Burlington, MA, USA). Iodoacetamid was used for alkylation. Trypsinolysis was performed using Trypsin Gold (Promega Corporation Madison, WI, USA), which was added to the protein in a ratio of 1/50.

### 4.5. Liquid Chromatography-Tandem Mass Spectrometry (LC-MS/MS) Based Proteomic Analysis

The obtained tryptic peptide mixture was analyzed using liquid chromatography-tandem mass spectrometry (LC-MS/MS) method based on a nano-HPLC Dionex Ultimate3000 system (Thermo Fisher Scientific, USA) and a TimsTOF Pro (Bruker Daltonics, Billerica, MA, USA) mass spectrometer. A packed emitter column (C18, 25 cm × 75 μm × 1.6 μm) (Ion Optics, Parkville, Australia) was used to separate peptides at a 400 nL/min flow rate gradient elution from 4% to 90% of phase B during 120 min. As a result, mobile phase A consisted of 0.1% formic acid in the water, and mobile phase B consisted of 0.1% formic acid in acetonitrile.

Mass spectrometric analysis was performed using the parallel accumulation—serial fragmentation (PASEF) acquisition method. An electrospray ionization (ESI) source was operated at 1500 V capillary voltage, 500 V endplate offset, and 3.0 L/min of dry gas at the temperature of 180 °C. The measurements were carried out in the m/z range from 100 to 1700 Th. The range of ion mobilities included values from 0.60 to 1.60 V s/cm2 (1/k_0_). The total cycle time was set to 1.16 s, and the number of PASEF MS/MS scans was set to 10. For low sample amounts, the total cycle time was set to 1.88 s.

The LC-MS/MS data obtained were analyzed using PEAKS Studio 8.5 and MaxQuant (version 1.6.7.0) according to the following parameters: parent mass error tolerance −30 ppm; fragment mass error tolerance −0.01 Da; enzyme-trypsin; missed cleavages-2; fixed modifications-Carbamidomethyl (C); variable modifications-Oxidation (M), Acetylation (N-term). The search was carried out using the SwissProt database. The false discovery rate threshold was set to 0.01.

### 4.6. Transmission Electron Microscopy

When delivered to the Earth, fixed bacterial samples were consecutively dehydrated in 50°, 70°, 96°, and 100° ethanol and embedded in LR White resin (SPI Supplies, Lakewood, WA, USA). Ultrathin sections were performed with the ultratome LKB III (LKB, Bromma, Sweden). The sections were contrasted with 1% uranyl acetate (Serva, USA) in 70° ethanol and with lead citrate (Serva Group LLS, Wichita Falls, TX, USA) and analyzed by the transmission electron microscope JEM 2100 Plus (Jeol, Tokyo, Japan) at 160 kV accelerating voltage. Bacterial lengths were calculated using the AMT Capture Engine Version 7.00 software. At least 10 fields of vision each including 5 to 10 bacterial cells were used to calculated the mean and standard deviation (SD).

### 4.7. Scanning Electron Microscopy

For SEM, fixed samples were prepared as previously described [16]. SEM was performed using the Camscan S2 microscope (Cambridge Scientific Instruments, Witchford, UK) in the secondary electron imaging (SEI) mode with a 10_nm optical resolution and an operating voltage of 20 kV. MicroCapture software (SMA, Moscow, Russia). The images were captured using MicroCapture software (SMA, Russia).

### 4.8. The Magnetic Experimental Setup and Calculation of Magnetic Force Affecting Bacteria

The magnetic systems used under the spaceflight and ground conditions were similar in their parameters. Each magnetic system included two NdFeB magnets with a material grade N38 and a remnant magnetization of 1.21 T. Magnets were ring shaped with the external diameter of 85 mm and the internal diameter of 20 mm and oriented to each other with the same poles. Being fixed in a such orientation, the magnets created non-homogenous static magnetic field with a very high gradient that was numerically calculated as gradB ≈ 2.2 T cm^−1^ in a working volume [48]. The magnetic field gradient created a magnetic force that pushed out diamagnetic particles such as bacteria into the area with the lowest magnetic field.

The projection of the magnetic force affecting bacteria on the z-axis was:

(1) $$Fm=\frac{\chi VB_{0}}{\mu_{0}}\frac{dB_{0}}{dz}$$

where *χ* is a magnetic susceptibility, *V* is a volume of the bacterial cell, *μ*_0_ is a vacuum permeability, *B_o_* is a magnetic flux density at a given point of the working volume, and $\frac{dB_{0}}{dz}$ is its gradient at the same point.

As soon as the paramagnetic medium was used, the coefficient K was introduced that considered relative magnetic permeabilities of the medium and cells:

(2) $$K=\frac{\mu_{p}-\mu_{f}}{\mu_{p}+2\mu_{f}}$$

where $\mu_{f}$ is relative magnetic permeability of the medium, $\mu_{p}$ in is relative magnetic permeability of the particle.

So,

(3) $$Fm=\frac{KVB_{0}}{\mu_{0}}\frac{dB_{0}}{dz}$$

The numerical calculations of *F_m_* have been performed as described in [48].

### 4.9. Statistical Analysis

Space samples were analyzed in duplicates as well as magnetic levitation samples. Other ground samples were replicated 3 to 5 times. The mean and standard deviation (SD) values were calculated from the entire data set where applicable. Statistical analysis was performed using one-way ANOVA with the post hoc Tukey’s test. The homogeneity of variance assumption was tested using Levene’s test. Statistical differences were considered significant when the *p*-value was <0.05.

## 5. Conclusions

Obtained results revealed a role of the glycosylate shunt, the methylglyoxal bypass, and Vitamin B12 transporter BtuB in *E. coli* prolonged survival, and the role of Ag43 in auto-aggregation under microgravity conditions. The magnetic force introduced into the spaceflight conditions strengthened cell response. This strengthening seems to be due to bacterial clustering in a small part of the total volume, in which the microgravity-driven absence of convectional flows reinforced oxygen and nutrition limitations. Ag43-driven auto-aggregation, up-regulation of BtuB, and activation of the glycosylate shunt might be required to provide effective utilization of a dead part of the population under microgravity and magnetic levitation conditions. Obtained results suggested that the model of magnetic levitation might be useful in simulated microgravity studies. Established effects of the magnetic force on bacteria cultivated in the space might be helpful for industrial purposes, i.e., bioproduction of certain compounds such as succinate or methylglyoxal. Taken together, our results demonstrated the potential of the magnetic force to modify bacterial behavior under spaceflight conditions, and that might be useful in future space research.

## Figures and Tables

**Table 1:** Morphologic characteristics of *E. coli* M17 grown under spaceflight and ground conditions.

Conditions	Length ^1^ of Preserved Cells ± SD (μm)
Ground 24 h	3.74 ± 0.31
Ground 144 h	3.27 ± 0.41
SF	3.0 ± 0.25
SF + MF	3.64 ± 0.42
Ground MF	1.92 ± 0.33

^1^ Mean values and SDs were calculated using at least 10 fields of vision which including 5 to 10 undamaged bacterial cells.

**Table 2:** Changes in protein composition compared to the ground control.

Protein Name	Log2 Expression Ratio				Protein Activity	Protein Function
	SF/144 h Control	SF + MF/144 h Control	ML/ 144 h Control	SF/ML		
Genetic information processing						
RS1	0.1 ^1^	n.d. ^2^	0.8 ^3^	−0.8	Ribosomal protein S1	Translation
RS6	0.7	1.2	−0.4	1.2	Ribosomal protein S6	Translation
Pnp	0.7	2.4	−0.5	1.2	Polynucleotide phosphorylase	tRNA processing
RpoB	−1.4	−2.6	−0.3	−1.1	Beta’-subunit RNA-polymerase	Transcription
Rho	0.9	1.0	−0.1	1.0	Transcription terminator	Regulation of transcription
Stress response						
KatG	1.0	1.8	0.25	0.8	Hydroperoxidase I	Oxidative stress response
WrbA	1.5	2.0	1.0	0.5	NADH:quinone oxidoreductase	Oxidative stress response
AhpC	0.2	1.1	−0.9	1.1	Alkyl hydroperoxide reductase subunit C	Oxidative stress response
DnaK	−1.6	−3.5	0.3	−1.9	Chaperone protein	Protein misfolding control
ClpB	−1.0	−3.1	1.1	−2.1	Chaperone protein	Protein misfolding control
ClpXP	−0.9	n.d.	2.6	−3.7	Chaperone protease	Protein misfolding control
Carbohydrate metabolism						
AceA	2.1	3.3	1.3	1.2	Isocitrate lyase	Glyoxylate shunt
Idh	−1.1	−4.1	1.9	−3.0	Isocitrate dehydrogenase	TCA
TdcE	−1.8	−3.8	0.2	−2.0	Pyruvate formate lyase 4	Glycolysis
PflB	−1.6	−2.5	0.1	−1.6	Pyruvate formate-lyase	Glycolysis
MgsA	0.7	2.5	−1.0	1.8	Methylglyoxal synthase	Synthesis of methylglyoxal
ElbB	1.3	n.d.	0.1	1.3	Glyoxalase III	Methylglyoxal detoxification
AckA	−0.8	n.d.	−1.2	0.5	Acetate kinase	Acetate metabolism
GlpA	0.2	1.6	−1.3	−1.4	Anaerobic Glyceraldehyde 3-phosphate dehydrogenase	Glycolysis/gluconeogenesis
Tkt1	0.1	1.6	−1.3	1.4	Transketolase	Pentose phosphate pathway
Surface structures						
BtuB	4.0	6.2	1.8	2.1	Cobalamin/cobinamide outer membrane transporter	Vitamin B12 transport
Ag43	1.4	1.3	1.3	0.1	Autotransporter adhesin Antigen 43	Control of autoaggregation
FliC	0.1	n.d.	3.8	−3.8	Flagella structural subunit	Motility

^1^ non-significant changes are shown in blue; ^2^ n.d.—not detected; ^3^ up-regulated proteins are shown in red, down-regulated proteins are shown by black.

## Data Availability

Data is contained within the article and Supplementary Material.

## Supplementary Materials

The following are available online at https://www.mdpi.com/article/10.3390/ijms23031837/s1.
