# Supplementary material for: Combined Impact of Magnetic Force and Spaceflight Conditions on Escherichia coli Physiology
Source: Int J Mol Sci. 2022 Feb 6;23(3):1837. doi: 10.3390/ijms23031837 (PMC8836844; doi:10.3390/ijms23031837)
Supplement: Supplementary file 1 [file ijms-23-01837-s001.zip › ijms-1531482-supplementary.pdf]

International Journal of Molecular Sciences

Calculation of Magnetic Force Affecting Bacteria under Magnetic Levitation Conditions

The magnetic force F_m affecting a bacterium in the stationary conditions of magnetic levitation can be easier evaluated from the following considerations. Three forces, which are the directed upward Archimedes force F_A, the directed toward gravity F_g, and the magnetic field F_m directed toward a local minimum of the magnetic field, act as the bacterial cell underground conditions. The bacterium was under magnetic levitation at the point where the resultant of these three forces taken along the vertical axe was zero,

$$F_g = F_A + F_m \quad (1)$$

then

$$F_m = F_g - F_A = g\, V\, (\rho_b - \rho_m) \quad (2)$$

where g is a gravity acceleration, V is a bacterial volume, ϱ_b is a bacterial specific density, and ϱ_m is a specific density of the medium. The *E. coli* cell volume is about 6 μm^3 (6 x 10^{-18} m^3). ϱ_m is close to the water density and will be considered as 1 kg × m^{-3}. As for bacterial specific density, the published values of microorganism specific density ranged between 1.05 ϱ_m up to 2 ϱ_m (). Then as it was determined from Equation 2, the range of magnetic force F_m was $F_m = (3 \div 60) \times 10^{-17}$ N.

To check these values, the magnetic force F_M that supported the aggregate formed by the bacteria and the extracellular matrix was calculated. The volume V of the aggregate was about 60 mm^3 (6 x 10^{-8} m^3). Using the same parameters of specific densities, the magnetic force $F_M = (3 \div 60) \times 10^{-7}$ N. The number of alive bacteria in the aggregate was 5 x 10^8 CFU. Supposing all bacteria has undergone the same F_m, we could suggest that F_M would be in the range of $(1.5 \div 30)\ 10^{-8}$ H. The discrepancy between values could be due to high presence of dead cells and matrix within the aggregate that increases the aggregate volume if compared to the sum of the bacterial volumes, as well as due to non-homogeneity of the magnetic flux all over the volume of the aggregate and corresponding changes in the magnetic force. Thus these calculations support the view that the range of magnetic force F_m affecting a single bacterial cell at the local point, where it is under conditions of magnetic levitation, was $F_m = (3 \div 60)\ 10^{-17}$ N.
