# Supplementary material for: Combined Impact of Magnetic Force and Spaceflight Conditions on Escherichia coli Physiology
Source: Int J Mol Sci. 2022 Feb 6;23(3):1837. doi: 10.3390/ijms23031837 (PMC8836844; doi:10.3390/ijms23031837)
Supplement: Supplementary file 1 [file ijms-23-01837-s001.zip › Table S1. Experiment scheme.pdf]

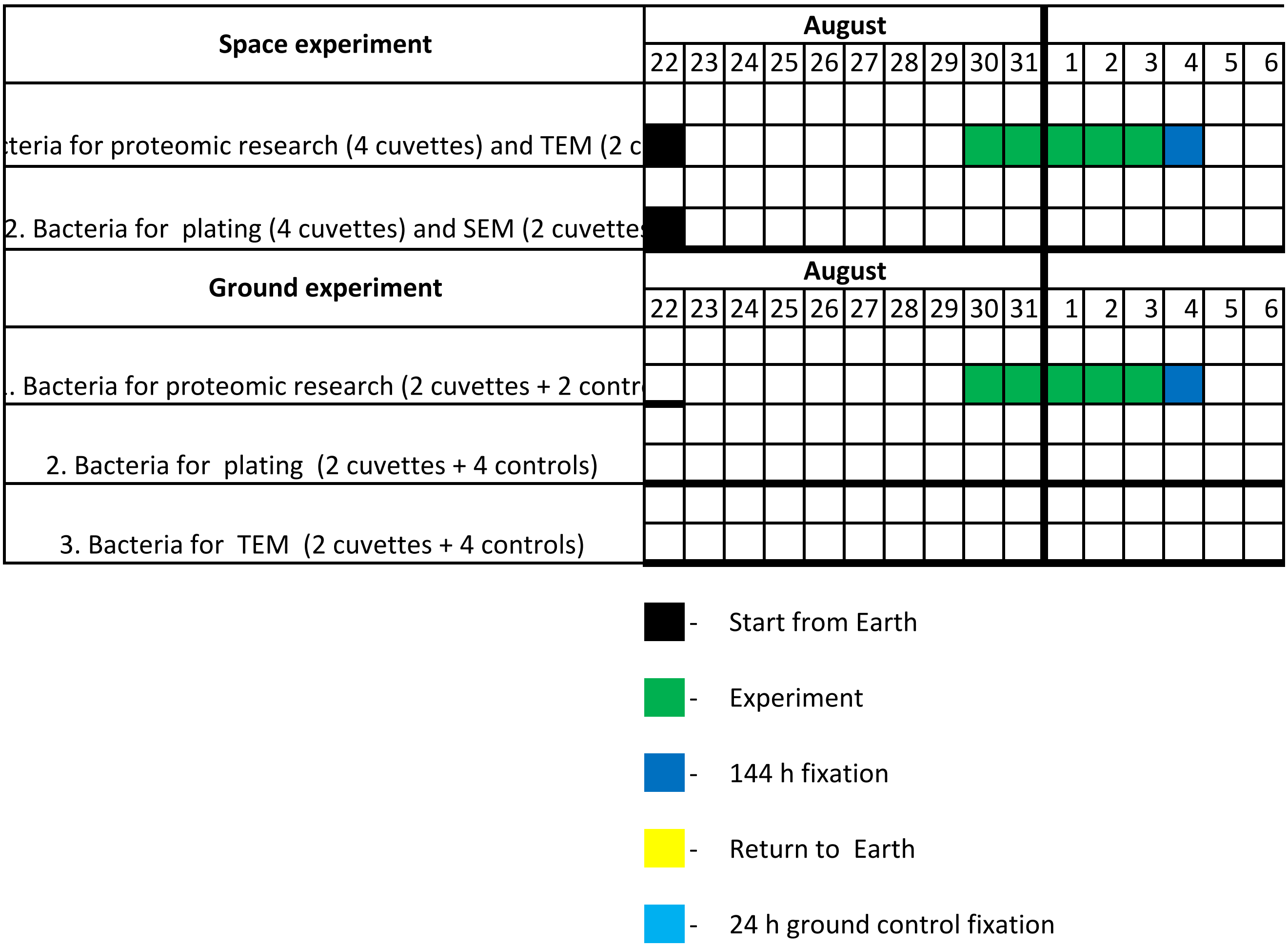
Space experiment
August
22 23 24 25 26 27 28 29 30 31 1 2 3 4 5 6
:teria for proteomic research (4 cuvettes) and TEM (2 c
2. Bacteria for plating (4 cuvettes) and SEM (2 cuvette
Ground experiment
August
22 23 24 25 26 27 28 29 30 31 1 2 3 4 5 6
. Bacteria for proteomic research (2 cuvettes + 2 contr
2. Bacteria for plating (2 cuvettes + 4 controls)
3. Bacteria for TEM (2 cuvettes + 4 controls)
- Start from Earth
- Experiment
- 144 h fixation
- Return to Earth
- 24 h ground control fixation

September																								Октябрь		
7	8	9	10	11	12	13	14	15	16	17	18	19	20	21	22	23	24	25	26	27	28	29	30	1	2	3

September																								Октябрь		
7	8	9	10	11	12	13	14	15	16	17	18	19	20	21	22	23	24	25	26	27	28	29	30	1	2	3
