# Supplementary figures and images for: Combined Impact of Magnetic Force and Spaceflight Conditions on Escherichia coli Physiology
Source: Int J Mol Sci. 2022 Feb 6;23(3):1837. doi: 10.3390/ijms23031837 (PMC8836844; doi:10.3390/ijms23031837)

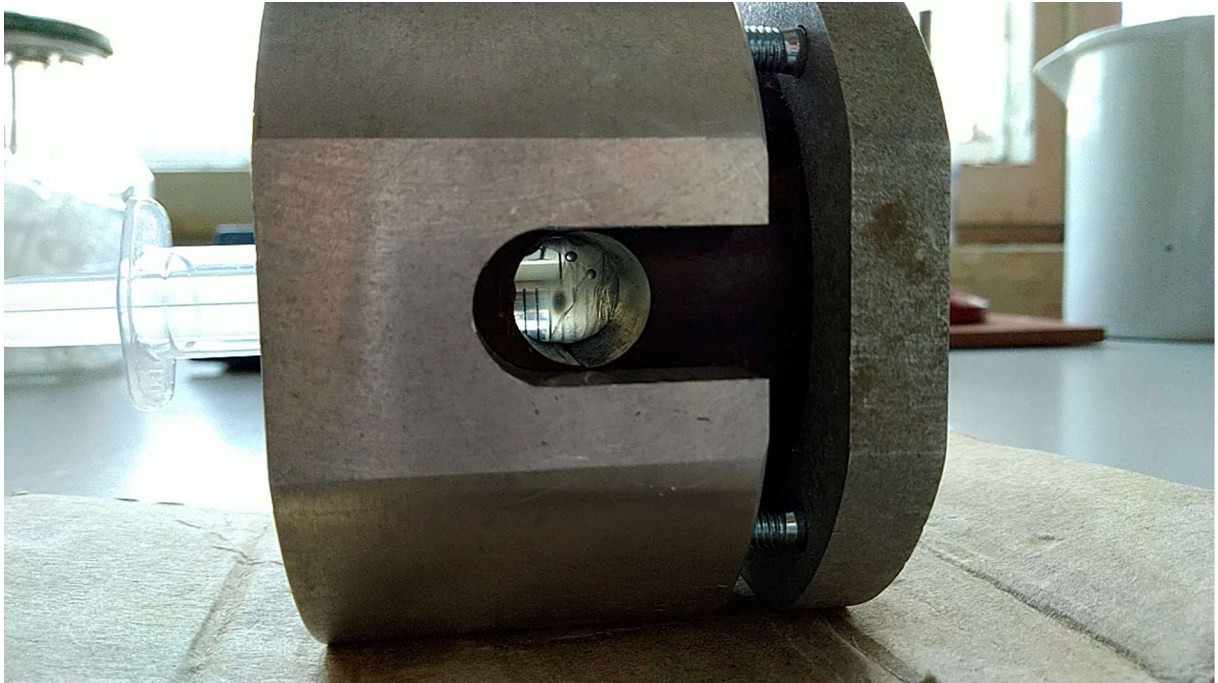

Supplement: Supplementary file 1 [file ijms-23-01837-s001.zip › Fig. S1.pdf]
